# Socioeconomic inequalities in hospitalizations for chronic ambulatory care sensitive conditions: a systematic review of peer-reviewed literature, 1990–2018

**DOI:** 10.1186/s12939-020-01160-0

**Published:** 2020-05-04

**Authors:** Lauren E. Wallar, Eric De Prophetis, Laura C. Rosella

**Affiliations:** Dalla Lana School of Public Health, University of Toronto, 155 College St, Toronto, ON M5T 3M7 Canada

**Keywords:** Ambulatory care sensitive conditions, Hospitalization, Socioeconomic status, Health inequalities, Observational studies, Systematic review

## Abstract

**Background:**

Hospitalizations for chronic ambulatory care sensitive conditions are an important indicator of health system equity and performance. Chronic ambulatory care sensitive conditions refer to chronic diseases that can be managed in primary care settings, including angina, asthma, and diabetes, with hospitalizations for these conditions considered potentially avoidable with adequate primary care interventions. Socioeconomic inequities in the risk of hospitalization have been observed in several health systems globally. While there are multiple studies examining the association between socioeconomic status and hospitalizations for chronic ambulatory care sensitive conditions, these studies have not been systematically reviewed. The objective of this study is to systematically identify and describe socioeconomic inequalities in hospitalizations for chronic ambulatory care sensitive conditions amongst adult populations in economically developed countries reported in high-quality observational studies published in the peer-reviewed literature.

**Methods:**

Peer-reviewed literature was searched in six health and social science databases: MEDLINE, EMBASE, PsycInfo, CINAHL, ASSIA, and IBSS using search terms for hospitalization, socioeconomic status, and chronic ambulatory care sensitive conditions. Study titles and abstracts were first screened followed by full-text review according to the following eligibility criteria: 1) Study outcome is hospitalization for selected chronic ambulatory care sensitive conditions; 2) Primary exposure is individual- or area-level socioeconomic status; 3) Study population has a mean age ± 1 SD < 75 years of age; 4) Study setting is economically developed countries; and 5) Study type is observational. Relevant data was then extracted, and studies were critically appraised using appropriate tools from The Joanna Briggs Institute. Results were narratively synthesized according to socioeconomic constructs and type of adjustment (minimally versus fully adjusted).

**Results:**

Of the 15,857 unique peer-reviewed studies identified, 31 studies met the eligibility criteria and were of sufficient quality for inclusion. Socioeconomic constructs and hospitalization outcomes varied across studies. However, despite this heterogeneity, a robust and consistent association between lower levels of socioeconomic status and higher risk of hospitalizations for chronic ambulatory care sensitive conditions was observed.

**Conclusions:**

This systematic review is the first to comprehensively identify and analyze literature on the relationship between SES and hospitalizations for chronic ambulatory care sensitive conditions, considering both aggregate and condition-specific outcomes that are common to several international health systems. The evidence consistently demonstrates that lower socioeconomic status is a risk factor for hospitalization across global settings. Effective health and social interventions are needed to reduce these inequities and ensure fair and adequate care across socioeconomic groups.

**Trial registration:**

PROSPERO CRD42018088727.

## Background

Socioeconomic inequalities in hospitalizations for ambulatory-care sensitive conditions (ACSCs), also known as avoidable or preventable hospitalizations, have been observed in multiple countries including Canada [1], the United States [2], England [3], France [4], Portugal [5], Australia [6], and others, where individuals of lower socioeconomic status (SES) are at higher risk of hospitalization compared to higher SES individuals. While the number and type of disease conditions considered sensitive to ambulatory or primary care differ [7], the underlying concept of ACSC hospitalizations is similar across health systems. ACSC hospitalizations refer to hospitalizations for certain disease conditions that are thought to be avoided if patients had received timely and effective primary care to prevent disease or disease exacerbations [8]. Hospitalization rates are routinely monitored by health systems as an indicator of health system performance at the nexus of primary and acute care [9, 10]. Socioeconomic inequalities in ACSC hospitalization rates may additionally indicate health system inequities along the care continuum that differentially affects the effectiveness of preventive and primary care received across socioeconomic groups [11] and may signal access barriers within a health system or other structural determinants of health. Thus, monitoring socioeconomic inequalities and understanding their root causes is important to improving health system performance and decreasing unnecessary hospitalizations.

SES is one of the most studied risk factors for ACSC hospitalizations. First described by Billings et al. in New York City [8], multiple studies have independently observed an association between SES and ACSC hospitalizations [12–16]. However, to our knowledge, these studies have not been systematically described. Previous narrative and systematic reviews of ACSC hospitalizations have focused on other risk factors including race and ethnicity [17, 18], primary care organization [19, 20], and other potential causes of geographic variation [21]. While the relationship between SES and hospitalizations for specific ACSCs such as asthma [22], congestive heart failure (CHF) [23], and chronic obstructive pulmonary disease (COPD) [24] have previously been reviewed, a comprehensive review considering the effect of SES on hospitalizations for ACSCs in aggregate and by condition has not been conducted. One particular challenge for this type of review is heterogeneity in the definitions of SES and ACSC hospitalizations [7]. To address this, this review adopts a more specific definition of SES and focuses only on chronic ACSCs that are consistent with the Canadian definition, namely angina, asthma, CHF, COPD, diabetes, epilepsy, and hypertension [9]. These conditions are common to other international health system definitions [10, 25, 26].

The objective of this review is to systematically identify and describe socioeconomic inequalities in chronic ACSC hospitalizations amongst adult populations in economically developed countries reported in high-quality observational studies published in the peer-reviewed literature.

## Methods

### Searches

MEDLINE, EMBASE, PsycInfo, CINAHL (Cumulative Index to Nursing and Allied Health Literature), ASSIA (Applied Social Sciences Index and Abstracts), and IBSS (International Bibliography of the Social Sciences) databases were searched from January 1, 1990 – July 31, 2018. Search strategies were iteratively developed and organized into three main blocks of terms by selecting and combining appropriate MeSH and keyword terms for 1) hospitalizations, 2) SES, and 3) ACSCs. Hospitalization terms were identified using the MeSH browser for relevant and related terms. SES terms were identified by consulting search strategies for systematic reviews published in high-quality peer-reviewed journals and registered systematic review protocols [27–35] as well as registered systematic review protocols [36, 37] where SES was the primary exposure of interest. The ACSC block of terms included both general ACSC terms (e.g. avoidable hospitalization) and terms for individual chronic ACSCs (iron-deficiency anemia, angina, atrial fibrillation and flutter, asthma, COPD, CHF, epilepsy and seizures, diabetes and diabetic complications, and hypertension). This ensured that studies of individual chronic ACSCs that did not specifically describe them as conditions sensitive to ambulatory care were still captured in the search. General terms were identified from keywords listed in abstracts of peer-reviewed articles related to ACSCs as well as published systematic reviews [21]. Previous Cochrane reviews of condition-specific outcomes were consulted to generate appropriate search strategies for each condition [38–51]. Exclusion filters for randomized controlled trials [52], countries classified as low-income to upper-middle-income economies according to the World Bank [53], articles published in languages other than English, articles published prior to 1990, and, where possible, publication sources other than peer-reviewed journals and books were then applied. Search strategies included known common alternative spellings (e.g. hospitalization vs hospitalisation), and were optimized for each database. See Additional file 1 for a search strategy exemplar.

### Study inclusion and exclusion criteria

Eligibility criteria for included studies are listed in Table 1.

**Table 1:** Study inclusion and exclusion criteria

Component	Explanation
Population	Inclusion: Study population has a mean age ± 1 SD < 75 years of age. If this criteria could not be evaluated due to missing data, the study was still included. Exclusion: • Pediatric studies were excluded during full-text review as both the concept and effect of SES on health outcomes differs in childhood relative to adulthood, and thus these studies were determined to be out of scope.
Exposure	Inclusion: Individual- or area-level SES defined as income, education, occupation, and social class in accordance with the WHO Commission on Social Determinants of Health conceptual framework. Exclusion: • SES was not the clear primary exposure of interest, including studies that did not include SES in their title or objectives, evaluated multiple predictors of interest (e.g. person and health system characteristics), or adjusted for SES as a confounding variable as these studies were not optimally designed to evaluate the effects of SES, decreasing interpretability of effect sizes. Exception: Studies that only included demographic and health status covariates as these are potential SES confounding variables, and SES effect sizes could be reasonably interpreted. • Only proxy measures for SES were used (e.g. car ownership, insurance status).
Outcome	Inclusion: Hospitalization, including emergency department visits, for chronic ACSCs. Both aggregate and condition-specific outcomes were included. Studies were included if aggregate outcomes included other ACSCs in addition to chronic ACSCs listed in this review. Exclusion: • Sole study outcome was length of stay or hospital readmissions as readmitted individuals were considered at greater risk of hospitalization relative to the general population. • Selected chronic ACSCs were narrowed during full-text review, excluding iron-deficiency anemia and atrial fibrillation and flutter, to be consistent with the Canadian definition (ie. angina, asthma, CHF, COPD, diabetes and diabetic complications, epilepsy and seizures, and hypertension). • Outcome was risk of hospitalization for an ACSC relative to a non-ACSC.
Study Setting	Inclusion: Countries with high-income economies.
Study Design	Inclusion: Observational (i.e. cross-sectional, case-control, or cohort). Exclusion: • Purely descriptive studies that did not clearly articulate the SES-ACSC relationship using measures of effect (i.e. risk ratio, rate ratio, odds ratio, hazard ratio, Relative Index of Inequality, Slope Index of Inequality).
Publication	Inclusion: • Written in the English language. • Published between January 1, 1990 to July 31, 2018. • Published in a peer-reviewed journal.

### Study selection procedure

Studies were selected for inclusion using a two-stage process. First, titles and abstracts were independently screened by LEW and EDP. Full texts of screened articles were then independently evaluated for inclusion by LEW and EDP. Any conflicts were resolved by discussion. If consensus could not be achieved, then a final decision was made in discussion with LCR. Reference lists of final articles included after quality assessment were hand searched by LEW. Study selection was managed using Covidence, an online tool for systematic review management.

### Data extraction strategy

A data extraction form was developed by LEW, piloted by LEW and EDP, and modified based on user findings and feedback. Data related to study characteristics, SES exposure, ACSC hospitalization outcome, and study results were extracted by LEW. Data extraction was managed using Microsoft Excel. See Additional file 2 for the data extraction form.

### Study quality assessment

Study quality assessment was conducted by LEW using appropriate The Joanna Briggs Institute Critical Appraisal Tools for a given observational study design [54]. Studies were appraised for selection and description of study participants, valid and reliable measurements of exposure and outcome, identification and control of potential confounders, and use of statistical methods. Studies that calculated rate ratios using standardized rates without additional adjustment for other confounders were separately synthesized as studies with minimal adjustment (ie. use of age- or age- and sex-standardized rates to calculate measures of association).

### Data synthesis and presentation

Included studies were qualitatively synthesized by type of SES exposure. Within each exposure type, results from studies with full adjustment for potential confounding variables were separately described from those with minimal adjustment (ie. use of age- or age- and sex-standardized rates to calculate measures of association). Given variation in measures of effect used, limiting cross-interpretation of effect sizes, the overall pattern of association between exposure and outcome were narratively described for each study. No additional analyses were conducted.

### Protocol and registration

PROSPERO CRD42018088727.

## Results

### Review statistics

**Fig. 1:**
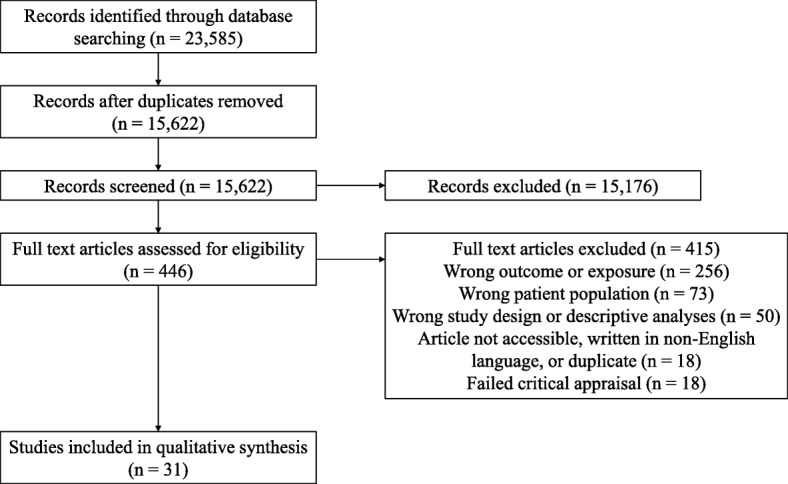
Study selection flow diagram

**Fig. 2:**
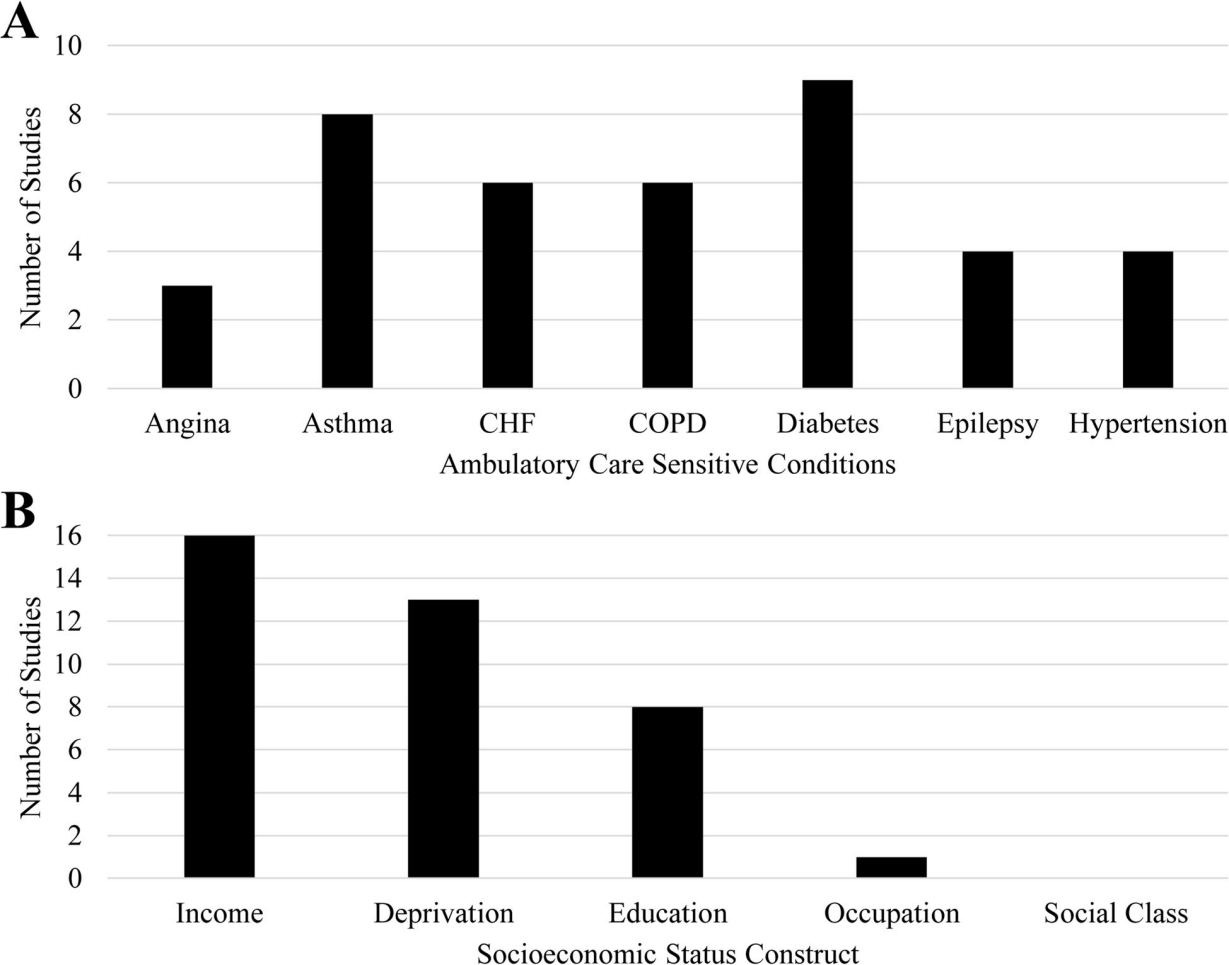
Number of included studies reporting on hospitalization for chronic ACSCs by condition (**a**) and operationalizing socioeconomic status along income, education, occupation, and class dimensions (**b**)

Of the 15,857 unique peer-reviewed studies identified, 31 studies met the eligibility criteria and were of sufficient quality for inclusion (Fig. 1) (Table 2). Study location was distributed across 11 different countries in North America (*n* = 14), Europe (*n* = 14), Oceania (*n* = 2), and Asia (*n* = 1). Both aggregate and condition-specific ACSC outcomes were reported (Fig. 2a), and multiple SES constructs were used including income, deprivation, education, and occupation (Fig. 2b). The majority of studies used group-level rather than individual-level SES exposure variables. All studies were either cohort or cross-sectional studies with almost half of the studies (*n* = 13) conducting a minimally adjusted analysis using standardized rates.

**Table 2:** Characteristics of peer-reviewed observational studies included in the systematic review

Citation	Country	Study Level	Study Type	Sample Size		Study Population	Exposure – SES		Outcome – ACSC admissions
				Numerator	Denominator	Disease Status	Variable	Level of observation	Chronic /Acute Conditions (Number of conditions if provided)
Agabiti, N. et al., 2009 [12]	Italy	Local / Regional	Cohort	9384 admissions	3,391,127 persons	General	Income	Group	Chronic Angina Asthma CHF COPD Diabetes Hypertension
Asaria, M. et al., 2016 [55]	England	National	Cross-sectional	Missing	32,482 neighbourhoods	General	Deprivation	Group	Chronic
Aube-Maurice, J. et al., 2012 [56]	Canada	State / Provincial	Cohort	17,688 pts	5,934,179 persons	Hypertension	Deprivation	Group	Hypertension
Bacon, S. et al., 2009 [57]	Canada	Local / Regional	Cross-sectional	Missing	781 persons	Asthma	Education	Individual	Asthma
Banham, D. et al., 2010 [58]	Australia	State / Provincial	Cross-sectional	54,573 pts. 79,424 admissions	Missing	General	Deprivation	Group	Acute, chronic, and vaccine-preventable [21]
Begley, C. et al., 2009 [59]	USA	Local / Regional	Cross-sectional	Missing	563 persons	Epilepsy	Income	Individual	Epilepsy
Bocour, A. et al., 2016 [60]	USA	Local / Regional	Cross-sectional	Missing	Missing	General	Income	Group	Angina Asthma CHF COPD Diabetes Hypertension
Booth, G. et al., 2003 [61]	Canada	State / Provincial	Cross-sectional	87,425 pts. 184,646 admissions	605,825 persons	Diabetes	Income	Group	Diabetes
Chen, P-C. et al., 2015 [62]	Taiwan	National	Cohort	1040 pts. 1298 admissions	57,791 persons	Diabetes	Income Education	Individual Group	Diabetes
Christensen, S. et al., 2011 [63]	Denmark	Local / Regional	Cohort	1473 pts. 1473 admissions	18,486 persons	General	Income Education	Individual Individual	CHF
Davies, S. et al., 2017 [64]	USA	State / Provincial	Cross-sectional	Missing	1778 counties	General	Income	Group	Chronic [5]
Disano, J. et al., 2010 [65]	Canada	National	Cross-sectional	Missing	46,173 dissemination areas	General	Deprivation	Group	Aggregate COPD Diabetes
Eisner, M. et al., 2011 [66]	USA	State / Provincial	Cohort	320 admissions	1202 persons	COPD	Income Education	Individual Individual	COPD
Fleetcroft, R. et al., 2017 [67]	England	National	Cross-sectional	Missing	32,482 neighbourhoods	General	Deprivation	Group	Diabetes
Govan, L. et al., 2012 [68]	Scotland	National	Cohort	4577 admissions	23,479 persons	Diabetes	Deprivation	Group	Diabetes
Gupta, R. et al., 2018 [69]	England	National	Cross-sectional	542,877 admissions	Missing	General	Deprivation	Group	Asthma
Jackson, R. et al., 2001 [70]	New Zealand	National	Cross-sectional	63,721 admissions	Missing	General	Deprivation	Group	Acute and Chronic
Lemstra, M. et al., 2006 [71]	Canada	Local / Regional	Cross-sectional	Missing	219,195 persons	General	Income	Group	COPD Diabetes
Li, X. et al., 2008 [72]	Sweden	National	Cohort	39,509 pts. 39,509 admissions	Missing	General	Income Education Occupation	Individual Individual Individual	Epilepsy
Li, X. et al., 2008 [58]	Sweden	National	Cohort	25,078 pts. 25,078 admissions	Missing	General	Education	Individual	Asthma
Lofqvist, T. et al., 2014 [14]	Sweden	Local / Regional	Cohort	18,956 pts. 29,841 admissions	1,431,338 persons	General	Income	Group	Acute and Chronic [12]
Macleod, M. et al., 2002 [73]	Scotland	Local / Regional	Cross-sectional	3340 admissions	Missing	General	Deprivation	Group	Epilepsy
Payne, R. et al., 2013 [15]	Scotland	National	Cohort	2037 pts	180,815 persons	General	Deprivation	Group	Acute, Chronic, and Vaccine-preventable [19]
Prescott, E. et al., 1999 [74]	Denmark	Local / Regional	Cohort	484 pts	14,223 persons	General	Income Education	Individual Individual	COPD
Quan, H. et al., 2013 [75]	Canada	State / Provincial	Cohort	Missing	3,531,089 persons	Hypertension	Income	Group	CHF
Roberts, S. et al., 2012 [76]	Wales	National	Cross-sectional	9986 pts. 12,740 admissions	3,000,000	General	Deprivation	Group	Asthma
Roos, L. et al., 2005 [16]	Canada	State / Provincial	Cohort	Missing	794,555 persons	General	Income	Group	Angina Asthma CHF Epilepsy
Shah, R. et al., 2011 [77]	USA	National	Cohort	663 pts. 663 admissions	26,160 persons	General	Income Education	Individual Individual	CHF
Sheringham, J. et al., 2017 [13]	England	National	Cross-sectional	Missing	316 local authority areas	General	Deprivation	Group	Chronic
Shulman, R. et al., 2018 [78]	Canada	State / Provincial	Cohort	Missing	8491 persons	Diabetes	Deprivation	Group	Diabetes
Walker, R. et al., 2013 [79]	Canada	State / Provincial	Cohort	2929 pts	786,529 persons	Hypertension	Income	Group	Hypertension

### Effect of SES on ACSC hospitalization outcomes

The majority of studies identified that lower SES was significantly associated with a higher risk of ACSC hospitalization, regardless of the SES exposure or ACSC hospitalization outcome measured (Table 3).

**Table 3:** Associations between socioeconomic status constructs and ambulatory care sensitive hospitalization outcomes in included studies (*n* = 31)

Citation	Measure of Association	ACSC	Effect Size; by model adjustment if provided	95% Confidence Interval	Direction of Association & Interpretation (+/−)
**Income - Fully Adjusted Analyses**					
Agabiti, N. et al., 2009 [12]	Rate Ratio (Lowest income quintile / Highest income quintile)	Chronic (*n* = 6) Angina Asthma CHF COPD Diabetes Hypertension	2.59 1.97 2.37 3.78 4.23 2.77 1.64	2.35–2.85 1.70–2.30 1.84–3.04 3.09–4.62 3.37–5.31 2.29–3.36 1.31–2.04	(−) As income decreases, hospitalization rate increases.
Begley, C. et al., 2009 [59]	Odds Ratio (Income < 100% of federal poverty level quartile / Income ≥400% of federal poverty level quartile	Epilepsy	Hospitalizations: 4.7 (Unadjusted) 2.9 (Adjusted for age, sex, and clinical characteristics) 0.8 (Additionally adjusted for treatment site) ER visits: 3.0 (Unadjusted) 2.2 (Adjusted for age, sex, and clinical characteristics) 0.5 (Additionally adjusted for treatment site)	1.4–15.9 0.9–9.9 0.2–3.3 1.6–5.7 1.1–4.3 0.2–1.4	(−) As income decreases, odds of ER visits and odds of hospitalization increases. Adjustment for treatment site mitigates income effect.
Booth, G. et al., 2003 [61]	Odds Ratio Unadjusted: (Lowest income quintile / Highest income quintile) Adjusted: Per decline in income quintile	Diabetes	1.43 (Unadjusted) 1.09 (Adjusted for age, sex, rurality, comorbidity, frequency of physician visits, continuity of care, physician speciality, and geographic region)	1.40–1.46 1.08–1.10	(−) As income decreases, odds of hospitalization or ED visits increases.
Chen, P-C. et al., 2015 [62]	Odds Ratio (Low income quartile / Highest income quartile)	Diabetes	2.89 (Adjusted for age, sex, time of diabetes diagnosis, comorbidities, participation in P4P program, education, and urbanization) 2.44 (Additionally adjusted for health care provider ownership and level)	2.19–3.83 1.81–3.30	(−) As income decreases, odds of hospitalization increases.
Christensen, S. et al., 2011 [63]	Hazard Ratio (High income tertile / Low income tertile)	CHF	0.67 (Female - Adjusted for age and time period) 0.66 (Male - Adjusted for age and time period)	0.51–0.89 0.42–0.66	(−) As income increases, hospitalization risk decreases.
Davies, S. et al., 2017 [64]	Rate Ratio (Highest decile of percent population below FPL / Lowest decile of percent population below FPL) (10th percentile of median income / 90th percentile of median income)	Chronic Chronic Asthma Asthma	1.91 (Percent below poverty line) 1.44 (Median household income) 1.50 (Percent below poverty line) 1.19 (Median household income)	1.78–2.04 1.35–1.53 1.39–1.62 1.11–1.27	(+) As percent below poverty line increases, ED visit risk increases. (−) As income decreases, ED visit risk increases.
Eisner, M. et al., 2011 [56]	Hazard Ratio (Low income tertile / High income tertile)	COPD	2.9 (Adjusted for age, sex, race, and education) 2.1 (Additionally adjusted for smoking history, occupational exposures, BMI, and co-morbidities) 1.5 (Additionally adjusted for COPD severity)	1.8–4.5 1.4–3.4 0.9–2.4	(−) As income decreases, hospitalization or ED visit risk increases.
Lofqvist, T. et al., 2014 [14]	Odds Ratio (Lowest income quintile / Highest income quintile)	Acute and chronic	Ages 18–64: 1.52 (Adjusted for age and sex) 1.12 (Additionally adjusted for marital status, country of birth, education, gainful employment, sickness benefit, and social assistance) Ages 65–79: 1.28 (Adjusted for age and sex) 1.06 (Additionally adjusted for marital status, country of birth, education, and social assistance)	1.44–1.60 1.06–1.19 1.21–1.36 1.00–1.13	(−) As income decreases, hospitalization rate increases.
Prescott, E. et al., 1999 [74]	Hazard Ratio (High income tertile / Low income tertile)	COPD	Male: 0.30 (Adjusted for age) 0.32 (Additionally adjusted for smoking status, inhalation, and duration of smoking) Female: 0.63 (Adjusted for age) 0.59 (Additionally adjusted for smoking status, inhalation, and duration of smoking)	0.20–0.45 0.21–0.49 0.40–1.01 0.37–0.95	(−) As income increases, hospitalization risk decreases.
Quan, H. et al., 2013 [75]	Hazard Ratio (Highest income quintile / Lowest income quintile)	CHF	0.72	0.71–0.73	(−) As income increases, hospitalization risk decreases.
Shah, R. et al., 2011 [77]	Hazard Ratio (Lowest income quartile / Highest income quartile)	CHF	3.43 (Unadjusted) 2.60 (Adjusted for age, race/ethnicity, marital status, and treatment assignments) 1.56 (Additionally adjusted for clinical characteristics, health behaviours, and insurance)	2.68–4.38 2.01–3.37 1.19–2.04	(−) As income decreases, hospitalization risk increases.
Walker, R. et al., 2013 [79]	Odds Ratio (Highest income quintile / Lowest income quintile)	Hypertension	0.59	0.51–0.68	(−) As income increases, odds of hospitalization decreases.
**Income - Minimally Adjusted Analyses**					
Bocour, A. et al., 2016 [60]	Rate Ratio (Very high poverty / Low poverty)	Angina Asthma CHF COPD Diabetes Hypertension	2.89 5.35 2.61 3.30 3.50 3.03	Missing	(+) As poverty increases, hospitalization rate increases.
Lemstra, M. et al., 2006 [71]	Rate Ratio (Low income / Affluent) (Dichotomous)	COPD Diabetes	1.53 12.86	0.88–2.67 5.42–30.51	(−) As income decreases, hospitalization rate increases.
Li, X. et al., 2008 [72]	Standardized Incidence Ratio (Low income tertile / All economically active persons	Epilepsy	1.13 (Males) 1.10 (Females)	1.11–1.15 1.07–1.12	(−) As income decreases, hospitalization rate increases.
Roos, L. et al., 2005 [16]	Rate Ratio (Lowest income quintile / Highest income quintile)	Angina Asthma CHF Epilepsy	1.39 2.90 1.73 2.98	1.21–1.58 2.50–3.37 1.58–1.92 2.17–4.36	(−) As income decreases, hospitalization rate increases.
**Education - Fully Adjusted Analyses**					
Bacon, S. et al., 2009 [57]	Risk Ratio (< 12 years of education / ≥ 12 years of education) Odds Ratio (< 12 years of education / ≥ 12 years of education)	Asthma	0.93 (Adjusted for age, sex, and asthma severity) 0.95 (Additionally adjusted for current smoking, BMI, and having a mood and/or anxiety disorder) 1.55 (Adjusted for age, sex, and asthma severity) 1.46 (Additionally adjusted for current smoking, BMI, and having a mood and/or anxiety disorder)	0.90–0.97 0.91–0.99 1.02–2.27 0.98–2.17	(+) As education decreases, risk of ED visits and hospitalizations increases. (−) As education decreases, odds of ED visits and hospitalizations increases.
Chen, P-C. et al., 2015 [62]	Odds Ratio (Lowest % of individuals with higher education quartile / Highest % of individuals with higher education quartile)	Diabetes	1.33 (Adjusted for age, sex, time of diabetes diagnosis, comorbidities, participation in P4P program, income, and urbanization) 1.32 (Additionally adjusted for health care provider ownership and level)	1.10–1.61 1.07–1.63	(−) As education decreases, odds of hospitalization increases.
Christensen, S. et al., 2011 [63]	Hazard Ratio (> 10 years of education tertile / < 8 years of education tertile)	CHF	0.50 (Female - Adjusted for age and time period) 0.53 (Male - Adjusted for age and time period) 0.52 (All - Adjusted for age, sex, and time period) 0.61 (Additionally adjusted for clinical characteristics, BMI, smoking, and physical inactivity)	0.37–0.69 0.42–0.66 0.43–0.63 0.50–0.73	(−) As education increases, hospitalization risk decreases.
Eisner, M. et al., 2011 [66]	Hazard Ratio (Less than high school education tertile / Post-secondary education completed tertile)	COPD	1.9 (Adjusted for age, sex, race, and education) 1.5 (Additionally adjusted for smoking history, occupational exposures, BMI, and co-morbidities) 1.1 (Additionally adjusted for COPD severity)	1.3–2.7 1.01–2.1 0.7–1.6	(−) As education decreases, risk of hospitalization or ED visit increases.
Prescott, E. et al., 1999 [74]	Hazard Ratio (> 11 years of education tertile / < 8 years of education tertile)	COPD	Male: 0.44 (Adjusted for age) 0.55 (Additionally adjusted for smoking status, inhalation, and duration of smoking) Female: 0.27 (Adjusted for age) 0.28 (Additionally adjusted for smoking status, inhalation, and duration of smoking)	0.27–0.72 0.34–0.90 0.11–0.65 0.12–0.69	(−) As education increases, hospitalization risk decreases.
Shah, R. et al., 2011 [77]	Hazard Ratio (Less than high school tertile / Post-secondary completed tertile)	CHF	2.01 (Unadjusted) 1.96 (Adjusted for age, race/ethnicity, marital status, and treatment assignments) 1.21 (Additionally adjusted for clinical characteristics, health behaviours, and insurance)	1.53–2.65 1.48–2.60 0.90–1.62	(−) As education decreases, hospitalization risk increases.
**Education - Minimally Adjusted Analyses**					
Li, X. et al., 2008 [72]	Standardized Incidence Ratio (≤ 9 years of education tertile / All economically active persons)	Asthma	1.03 (Male) 1.05 (Female)	1.01–1.05 1.03–1.08	(−) As education decreases, hospitalization rate increases.
Li, X. et al., 2008 [72]	Standardized Incidence Ratio (≤ 9 years of education tertile / All economically active persons)	Epilepsy	1.06 (Male) 1.06 (Female)	1.04–1.08 1.04–1.08	(−) As education decreases, hospitalization rate increases.
**Occupation - Minimally Adjusted Analyses**					
Li, X. et al., 2008 [72]	Standardized Incidence Ratio (Unskilled workers / All economically active persons)	Epilepsy	1.04 (Male) 1.01 (Female)	1.02–1.06 0.99–1.03	(−) As occupation decreases, hospitalization rate increases.
**Deprivation - Fully Adjusted Analyses**					
Aube-Maurice, J. et al., 2012 [56]	Risk Ratio (Most deprived quintile / Least deprived quintile)	Hypertension	Males: 1.29 (Material deprivation) 1.14 (Social deprivation) Females: 1.60 (Material deprivation) 1.04 (Social deprivation)	1.18–1.40 1.05–1.24 1.43–1.79 0.93–1.16	(+) As deprivation increases, hospitalization risk increases.
Govan, L. et al., 2012 [68]	Odds Ratio (Most deprived quintile / Least deprived quintile)	Diabetes	2.82	2.33–3.42	(+) As deprivation increases, odds of hospitalization increases.
Gupta, R. et al., 2018 [69]	Rate Ratio (Most deprived quintile / Least deprived quintile)	Asthma	3.34 (Ages 5–44) 2.01 (Ages 45–74)	3.30–3.38 1.98–2.05	(+) As deprivation increases, hospitalization rate increases.
Payne, R. et al., 2013 [15]	Odds Ratio (Most deprived quintile / Least deprived quintile)	Acute and chronic	2.84 (Unadjusted) 1.98 (Adjusted for age, sex, multimorbidity, and mental health condition)	2.40–3.37 1.63–2.41	(+) As deprivation increases, odds of hospitalization increases.
Shulman, R. et al., 2018 [78]	Rate Ratio (Most deprived quintile / Least deprived quintile)	Diabetes	(Hospitalizations) (ER visits)	Values not reported. See Fig. 2 in article.	(+) As deprivation increases, hospitalization and ER visit rate increases.
**Deprivation - Minimally Adjusted Analyses**					
Asaria, M. et al., 2016 [55]	RII SII	Chronic	1.06 6.07	1.04–1.07 5.97–6.16	(+) As deprivation increases, hospitalization risk increases.
Banham, D. et al., 2010 [58]	Rate ratio (Most disadvantaged quintile / Least disadvantaged quintile)	Acute, chronic, and vaccine-preventable	2.5	2.5–2.5	(+) As deprivation increases, hospitalization rate increases.
Disano, J. et al., 2010 [65]	Rate Ratio (Low SES tertile / High SES tertile)	Aggregate COPD Diabetes	2.6 2.7 3	Missing Missing Missing	(+) As deprivation increases, hospitalization rate increases.
Fleetcroft, R. et al., 2017 [67]	RII SII	Diabetes	1.18 84.25	1.15–1.22 81.62–86.88	(+) As deprivation increases, hospitalization risk increases.
Jackson, R. et al., 2001 [70]	Rate Ratio (Most deprived 10% / Least deprived 40%)	Aggregate	2.3	Missing	(+) As deprivation increases, hospitalization rate increases.
Macleod, M. et al., 2002 [73]	Rate Ratio (Most deprived septile / Least deprived septile)	Epilepsy	3.30	Missing	(+) As deprivation increases, hospitalization rate increases.
Roberts, S. et al., 2012 [76]	Rate Ratio (Most deprived quintile / Least deprived quintile)	Asthma	2.48	2.34–2.62	(+) As deprivation increases, hospitalization rate for severe asthma increases.
Sheringham, J. et al., 2017 [13]	SII	Aggregate	5.98	Missing	(+) As deprivation increases, hospitalization risk increases.

#### Income

Of the 12 studies with full adjustment for confounding variables, 11 studies found that lower income was associated with a higher risk or rate of ACSC hospitalizations in their fully adjusted models with 10 of these studies reporting significant effects. In contrast, one study of the effect of income on epilepsy hospitalizations and ER visits found that lower income decreased odds of hospitalizations and ER visits after adjusting for treatment site, but the effect was not significant [59]. Four minimally adjusted analyses also found that lower income significantly increased risk of hospitalization with only one study reporting a non-significant relationship between income and rate of COPD hospitalizations [71].

#### Education

All six fully adjusted studies found that lower education is associated with higher risk of ACSC hospitalization with three of these studies reporting significant associations. One study of the effect of education on asthma hospitalizations or ED visits reported conflicting results when using Poisson versus logistic regression [57]. Two minimally adjusted analyses similarly found significant associations between lower education and increased incidence of asthma and epilepsy hospitalizations.

#### Occupation

The sole minimally adjusted study of the effect of occupational class on incidence of epilepsy hospitalizations observed that lower occupational class is weakly associated with higher ACSC hospitalization rates [72].

#### Deprivation

All five fully adjusted studies observed that higher deprivation is associated with higher risk or rate of ACSC hospitalizations. Eight minimally adjusted analyses reported the same findings, four of which were significant and four could not be determined due to missing confidence interval information.

### Quality assessment

Forty-nine studies were critically appraised on characteristics that could introduce different biases including study population selection, exposure and outcome measurement, loss to follow up (cohort studies), identification and control of potential confounding variables, and usage of appropriate statistical methods. Of the 49 studies, 18 were excluded due to poor quality assessments. A number of studies (*n* = 11) used linear regression to model rate outcomes. This can be seen as inappropriate since linear regression assumes an incorrect error distribution for rate outcomes, which violates model assumptions. Two studies used other inappropriate methodologies, including choice of an outdated standard population for standardizing hospitalization rates and treating longitudinal data of the same study population as independent observations. Three studies lacked sufficient methodological information required for critical appraisal. One study used an appropriate regression model but selected variables using backward stepwise selection, which can artificially select for variables with small *p*-values and yield a final model that does not appropriately adjust for SES confounding variables. Lastly, one cohort study had a high risk of loss-to-follow up, which can result in high risk of bias. Study specific quality assessments can be found in Additional files 3 and 4.

Of the 31 included studies, 15 studies conducted analyses that minimally adjusted for potential SES confounding variables (ie. use of age- or age- and sex-standardized rates to calculate measures of association) and thus had higher risk of residual confounding of their reported estimates. The remaining 18 studies were of sufficient quality with low risk of bias. However, potential biases in these studies were possible. Studies inferring individual-level SES using group-level exposure information (*n* = 11) are at risk of exposure misclassification. While most of these studies identified hospitalizations using registries, three studies used self-reported information which may be less accurate and subject to recall bias due to varying recall ability of study participants for when and why they were hospitalized. Lastly, studies varied in identification and control of potential confounding variables. While some studies presented sequentially adjusted models, others only presented fully adjusted models. Despite this variation, patterns of association within and across studies generally remained the same, although the magnitude of effect was attenuated after adjustment.

### Evidence of effectiveness

In this review, almost all studies reported a relationship between low SES and high ACSC hospitalization outcomes with lower SES associated with higher risk of ACSC hospitalization outcomes across different SES exposures, aggregate and condition-specific outcomes, and study level. Two studies reporting a positive effect in their fully adjusted models were both local/regional studies of condition specific ACSC outcomes with limited sample sizes (< 800 persons), and thus are of lower impact. Strong consistency of results provides evidence of an association between SES and ACSC hospitalizations with lower SES individuals at greater risk of hospitalization compared to higher SES individuals.

### Discussion

### Summary of key findings

This systematic review is the first to comprehensively identify and analyze peer-reviewed literature on the relationship between SES and chronic ACSC hospitalizations, considering both aggregate and condition-specific outcomes that are common to a number of international health systems that utilize ACSC hospitalization rates as an indicator of health system performance. The review determined that there is an increased risk of chronic ACSC hospitalizations associated with lower levels of SES and this finding is robustly observed across different SES exposure definitions, aggregate and condition-specific hospitalization outcomes, local to national observational levels, and varied geographic locations. Studies vary in their methodological approaches including identification and control of confounding variables and definition of outcomes. Inappropriate use of regression techniques to model hospitalization rate outcomes is not uncommon and limits interpretation of some study findings.

### Strengths and limitations

This review specifically focused on chronic ACSC hospitalizations that are commonly measured in other international health systems that monitor ACSC hospitalization rates, increasing relevance of study findings. Distinguishing chronic from acute and vaccine-preventable ACSC hospitalizations is important as the impact of SES on hospitalization rates for these conditions likely differs between these groupings of conditions and should be separately summarized [80]. Chronic ACSC hospitalizations are of importance to both population health and primary health care as prevalence of chronic ACSCs, and correspondingly, size of populations at greater risk of hospitalization, is increasing within countries. A greater understanding of upstream risk factors, such as SES, is needed to help address this increasingly important population health, health system, and health equity outcome.

Our methodological approach had a number of strengths. First, the search strategy incorporated both general and condition-specific ACSC terms to increase sensitivity of the search as studies of condition-specific outcomes will not necessarily use generic ACSC terms (e.g. avoidable, preventable, ambulatory care sensitive) in their titles and abstracts. Consultation and usage of high-quality, published search strings for each condition also helped to successfully retrieve articles relating to these conditions. Use of both health and social science databases further ensured that studies of SES and ACSC hospitalization outcomes were identified. Lastly, this review specifically focused on studies where SES was the primary exposure of interest. SES is a very common independent variable that is included in studies of ACSC hospitalization outcomes. However, we distinguished between usage of SES as an exposure variable versus an adjustment or confounding variable. While this possibly decreased some eligible studies, this allowed us to more specifically interpret reported associations between SES and chronic ACSC hospitalizations by reviewing studies designed to explicitly assess this relationship. In addition, studies that were ambiguous on the role of SES as an exposure also tended to be of lower quality.

This review had several limitations that are important to acknowledge. First, this review was limited to peer-reviewed literature, potentially missing grey literature evaluating the impact of SES on chronic ACSC hospitalizations. Second, we opted for more specific definitions of both SES and chronic ACSC hospitalizations in order to be more precise in the interpretation and aid in synthesis of results, potentially excluding studies using alternative SES definitions (e.g. insurance status, home ownership, car ownership). We specifically chose SES constructs that are commonly used across health systems in high-income economies to increase relevance. System-specific measures (e.g. insurance status in the US health system) and proxy measures of wealth were considered less relevant given the scope of this review. Likewise, a limited number of chronic ACSC hospitalizations were considered and did not include all possible chronic ACSCs evaluated by international health systems. We specifically chose conditions consistent with the Canadian definition that is used for health system performance monitoring. Further, these conditions are also measured by other similar health systems, and thus this limitation is not expected to greatly impact the relevance of this review in an international context. Importantly, we did not exclude studies using aggregate ACSC hospitalization outcomes that included both chronic and other ACSC conditions. While interpretation of studies using mixed aggregate outcomes was reduced, it more importantly prevented bias towards selection of studies evaluating condition-specific rather than aggregate outcomes. Lastly, we were unable to conduct a meta-analysis given the extensive heterogeneity in outcome definitions, exposure definitions, and measures of effect used.

### Heterogeneity in studies of SES and chronic ACSC hospitalizations

There is inherent heterogeneity in how both SES and chronic ACSC hospitalizations are defined that precludes additional synthesis of results beyond narrative syntheses. In this review, SES was defined using multiple constructs. Even within a given construct, multiple definitions were used. For example, income constructs included area-level household income, individual-level household income, and percentage below federal poverty line. Deprivation was measured using multiple context-specific indices including Index of Multiple Deprivation, Welsh Index of Multiple Deprivation, Scottish Index of Multiple Deprivation, and INSPQ Deprivation Index that measure both similar and unique aspects of deprivation, making cross-national generalizations difficult [81]. While education was most consistently defined, the number and meaning of categorical levels varied between studies. Likewise, definitions of chronic ACSC hospitalizations also varied including diagnostic codes used to identify conditions, inclusion of emergency department visits, hospital transfers, and hospital readmissions, and usage of age thresholds. Although we found consistent associations across different exposure and outcome definitions, this heterogeneity prevented additional meta-analyses of the effect of SES on chronic ACSC hospitalizations.

### Considerations for future studies

There are a number of findings that have emerged from this review that are useful to consider for future research of SES and ACSC hospitalizations. Firstly, we note several challenges with the reporting of the effect of SES on ACSC hospitalization risk in the included observational studies. We recommend that researchers clarify the role that SES is playing in the analysis (i.e. as a primary exposure or not), which will impact how it is handled in the analysis. If SES is one of many covariates of interest, studies should publish models that sequentially and transparently adjust for different factors (e.g. demographic, socioeconomic, behavioural, health system) to facilitate interpretation of SES variables. If SES is a priori considered a confounding variable, then studies should consider how the SES effect size is interpreted as the model is not optimized for SES as an exposure. The ambiguity around the role of SES in the analysis of the included studies made summarizing the literature challenging.

Second, we recommend clarity in considering hospitalization outcomes. We found considerable variation in how hospitalization outcomes were defined. Specifically, there was confusion regarding whether all-cause or cause-specific hospitalizations are measured, usage of diagnostic codes including whether primary only or primary and secondary codes were used, and whether readmissions were included. Further, we found variability in how studies described their comparator outcome group; specifically, indicating whether this group includes non-hospitalized individuals, individuals who are hospitalized but for a non-ACSC condition, or individuals who are hospitalized once for an ACSC condition. Lack of clarity in the comparison group prevents the ability to do meta-analysis in future reviews.

Third, we recommend more consistent reporting of study population characteristics. This was commonly missing, particularly in cross-sectional studies. These characteristics include: number of persons hospitalized, number of hospitalizations, age and sex distribution, and denominator information. Additional methodological considerations include using Poisson or negative binomial regression versus linear regression to model rate outcomes and avoiding use of stepwise selection of variables that may eliminate important confounders and affect standard errors. Stratification of results by age groups (e.g. pediatric, adult, and elderly) was also not commonly done but may be useful as the concept of both SES and avoidable hospitalizations are different within each of these groups.

With respect to future areas of research, we note that additional research was lacking in three important areas. First, while this review focused on chronic conditions, systematic reviews of SES as a risk factor for ACSC hospitalizations for acute and vaccine-preventable conditions were much more limited and would be needed for a more comprehensive understanding [80]. Second, more studies that specifically focus on elucidating the mechanisms between SES and ACSC hospitalizations. This includes the potential of SES as an indirect risk factor that is mediated through more proximal factors. Lastly, there are few studies that examine the delivery and impact of interventions for policies that may mitigate the impact of lower SES on risk of hospitalization by either directly improving SES conditions or through healthcare approaches.

## Conclusions

This systematic review found a robust and consistent association between lower levels of SES and higher risk of chronic ACSC hospitalizations. Given the population health and primary health care importance of reducing these hospitalizations, an understanding and focus on upstream risk factors such as SES is warranted. The consistent and persistent SES inequalities observed in this review suggest that much work remains to reduce hospitalization risk among lower SES individuals. Additional evidence is needed on effective interventions for reducing overall hospitalization rates and reducing the inequality gap between different SES groups.

## Supplementary information

**Additional file 1.** EMBASE Search Strategy. Search strategy used to identify articles in the EMBASE database including search terms, search strings, and filters.

**Additional file 2.** Data Extraction Form. Form used to extract data from eligible full-text articles.

**Additional file 3. **Critical Appraisal of Included Articles (*n* = 31) using The Joanna Briggs Institute Critical Appraisal Tools. Study quality assessment results for each article included in the systematic review from eligible full-text articles.

**Additional file 4. **Critical Appraisal of Articles Excluded after Appraisal (*n* = 18) using The Joanna Briggs Institute Critical Appraisal Tools. Study quality assessment results for each article excluded upon appraisal from eligible full-text articles.

## Data Availability

Data sharing is not applicable to this article as no datasets were generated or analyzed during the current study.

## Abbreviations

ACSC: Ambulatory care sensitive condition
CHF: Congestive heart failure
COPD: Chronic obstructive pulmonary disease
INSPQ: Institut national de santé publique du Québec
SES: Socioeconomic status
