# Supplementary material for: Socioeconomic inequalities in hospitalizations for chronic ambulatory care sensitive conditions: a systematic review of peer-reviewed literature, 1990–2018
Source: Int J Equity Health. 2020 May 4;19:60. doi: 10.1186/s12939-020-01160-0 (PMC7197160; doi:10.1186/s12939-020-01160-0)
Supplement: Supplementary file 1 — Additional file 1. EMBASE Search Strategy. Search strategy used to identify articles in the EMBASE database including search terms, search strings, and filters. [file 12939_2020_1160_MOESM1_ESM.docx]

Additional File 1. EMBASE Search Strategy

| **#** | **Searches** |
| --- | --- |
| 1 | HOSPITALIZATION/ |
| 2 | Patient Admission/ |
| 3 | Patient Readmission/ |
| 4 | Length of Stay/ |
| 5 | (hospitali* or rehospitali* or re-hospitali*).ti,ab,kw. |
| 6 | (patient? adj5 (admission* or readmission* or re-admission* or admit* or readmit* or re-admit* or visit* or revisit* or re-visit* or referral*)).ti,ab,kw. |
| 7 | ((hospital or inpatient or outpatient or emergency) adj5 (admission* or readmission* or re-admission* or admit* or readmit* or re-admit* or visit* or revisit* or re-visit* or referral* or use? or usage? or utili#e? or utili#ation? or care or service? or department?)).ti,ab,kw. |
| 8 | (hospital stay? or stay length? or (length? of adj2 stay)).ti,ab,kw. |
| 9 | LOS.ti,ab,kw. |
| 10 | or/1-9 [**Hospitalization] |
| 11 | Healthcare Disparities/ |
| 12 | Health Equity/ |
| 13 | Health Status Disparities/ |
| 14 | Social Determinants of Health/ |
| 15 | Social Marginalization/ |
| 16 | exp Socioeconomic Factors/ |
| 17 | Vulnerable Populations/ |
| 18 | ((health or healthcare or health-care or income or wealth or education* or occupation* or vocation*) adj5 (disadvantag* or disparit* or equit* or inequalit* or inequit*)).ti,ab,kw. |
| 19 | ((social or socially or socioeconomic* or socio-economic* or sociodemographic* or socio-demographic*) adj2 (depriv* or difference? or disadvantag* or discriminat* or disparit* or equalit* or equit* or exclude? or exclusi* or include? or inclusi* or inequalit* or inequit* or marginali*)).ti,ab,kw. |
| 20 | ((socioeconomic* or socio-economic* or sociodemographic* or socio-demographic*) adj2 (characteristic? or class* or condition? or determinant? or factor? or gap? or gradient? or group* or hierarch* or indicator? or level? or population? or position? or standard? or standing? or status or strata or stratum or stratification? or variabilit* or variation?)).ti,ab,kw. |
| 21 | SES.ti,ab,kw. |
| 22 | (social adj2 (determinant? or gradient?)).ti,ab,kw. |
| 23 | ((upper or middle or lower or working or social) adj2 (class or classes)).ti,ab,kw. |
| 24 | (income? or impoveri* or indigenc* or indigent or non-poor or non poor or poverty or wealth).ti,ab,kw. |
| 25 | ((individual* or personal or famil* or household? or house-hold? or house hold?) adj2 (earning? or wage? or consumption* or saving* or revenue* or pay or salar* or poor)).ti,ab,kw. |
| 26 | ((socioeconomic* or socio-economic* or sociodemographic* or socio-demographic* or income or wealth or occupation* or vocation*) and education*).ti,ab,kw. |
| 27 | ((socioeconomic* or socio-economic* or sociodemographic* or socio-demographic* or income or wealth or education*) and (occupation* or vocation*)).ti,ab,kw. |
| 28 | ((depriv* or disadvantag* or discriminat* or marginali* or underserv* or under-serv* or under serv* or vulnerab*) adj3 (individual? or patient? or family or families or group? or population?)).ti,ab,kw. |
| 29 | or/11-28 [**SES] |
| 30 | Anemia, Iron-Deficiency/ |
| 31 | Anemia, Hypochromic/ |
| 32 | (an?emi* or chloros#s).ti,ab,kw. |
| 33 | ((iron adj3 deficien*) or iron-deficien*).ti,ab,kw. |
| 34 | Plummer-Vinson Syndrome/ |
| 35 | ((kelly or kelly's or kellys or paterson or paterson's or patersons or paterson-kelly or paterson-brown-kelly or kelly-paterson or plummer vinson or plummer-vinson or glossitis or faber or waldenstrom-kjellberg) adj2 syndrome).ti,ab,kw. |
| 36 | (sideropenic adj (dysphagia or nasopharyngopathy)).ti,ab,kw. |
| 37 | or/30-36 [**Iron-deficiency anemia] |
| 38 | exp Angina Pectoris/ |
| 39 | angina*.ti,ab,kw. |
| 40 | stenocardia*.ti,ab,kw. |
| 41 | angor pectoris.ti,ab,kw. |
| 42 | or/38-41 [**Angina Pectoris] |
| 43 | Coronary Disease/ |
| 44 | Coronary Artery Disease/ |
| 45 | Coronary Stenosis/ |
| 46 | Coronary Thrombosis/ |
| 47 | (coronary arter* and stenos*).ti,ab,kw. |
| 48 | coronary steno*.ti,ab,kw. |
| 49 | coronary atheroscleros*.ti,ab,kw. |
| 50 | coronary arterioscleros*.ti,ab,kw. |
| 51 | coronary thrombos#s.ti,ab,kw. |
| 52 | (coronary adj5 disease*).ti,ab,kw. |
| 53 | CAD.ti,ab,kw. |
| 54 | or/43-53 [**Coronary Disease] |
| 55 | exp Myocardial Ischemia/ |
| 56 | exp Heart Failure/ |
| 57 | Heart Diseases/ |
| 58 | ((myocardial or myocardium or subendocardial or transmural or cardiac or cardial or coronary or heart) adj2 (infarct* or postinfarct* or hypoxi* or anoxi* or failure* or decompensation or insufficien*)).ti,ab,kw. |
| 59 | (heart disease* or coronary disease* or IHD or CIHD).ti,ab,kw. |
| 60 | (myocardial d#sfunction or angina or stenocardia).ti,ab,kw. |
| 61 | ((ischemi* or ischaemi*) adj2 (myocardium or myocardial or heart or coronary or cardiac or cardial or subendocardial or cardiomyopath*)).ti,ab,kw. |
| 62 | ((end stage or endstage) adj cardiomyopath*).ti,ab,kw. |
| 63 | ((artery occlusion* or artery disease* or arterioscleros* or atheroscleros*) adj2 coronary).ti,ab,kw. |
| 64 | ((heart or cardiac or cardial or myocardium or myocardial) adj3 (repair* or reparation or improve* or regenerat*)).ti,ab,kw. |
| 65 | or/55-64 [**Myocardial Ischemia] |
| 66 | or/42,54,65 [**Angina] |
| 67 | Atrial Fibrillation/ |
| 68 | Atrial Flutter/ |
| 69 | Tachycardia, Ectopic Atrial/ |
| 70 | ((atrial or atrium or auricular) adj2 (fibrillat* or flutter*)).ti,ab,kw. |
| 71 | ((atrial or atrium or auricular) adj2 (tachycardia* or tachyarrhythmia*)).ti,ab,kw. |
| 72 | or/67-71 [**Atrial fibrillation and flutter] |
| 73 | exp Asthma/ |
| 74 | asthma*.ti,ab,kw. |
| 75 | (antiasthma* or anti-asthma*).ti,ab,kw. |
| 76 | Respiratory Sounds/ |
| 77 | wheez*.ti,ab,kw. |
| 78 | Bronchial Spasm/ |
| 79 | bronchospas*.ti,ab,kw. |
| 80 | (bronch* adj3 spasm*).ti,ab,kw. |
| 81 | exp Bronchoconstriction/ |
| 82 | bronchoconstrict*.ti,ab,kw. |
| 83 | (bronch* adj3 constrict*).ti,ab,kw. |
| 84 | Bronchial Hyperreactivity/ |
| 85 | Respiratory Hypersensitivity/ |
| 86 | ((bronchial* or respiratory or airway* or lung*) adj3 (hypersensitiv* or hyperreactiv* or allerg* or insufficien*)).ti,ab,kw. |
| 87 | ((dust or mite*) adj3 (allerg* or hypersensitiv*)).ti,ab,kw. |
| 88 | or/73-87 [**Asthma] |
| 89 | exp Bronchitis/ |
| 90 | bronchit*.ti,ab,kw. |
| 91 | exp Pulmonary Disease, Chronic Obstructive/ |
| 92 | (chronic obstructive pulmonary disease* or chronic obstructive lung disease* or chronic obstructive airway disease*).ti,ab,kw. |
| 93 | Lung Diseases, Obstructive/ |
| 94 | (copd or coad or cobd or aecb).ti,ab,kw. |
| 95 | exp Emphysema/ |
| 96 | emphysema*.ti,ab,kw. |
| 97 | (chronic* adj3 bronchiti*).ti,ab,kw. |
| 98 | (obstruct* adj3 (pulmonar* or lung* or airway* or airflow* or bronch* or respirat*)).ti,ab,kw. |
| 99 | ((lung* or thorax) adj3 hyperlucen*).ti,ab,kw. |
| 100 | exp Bronchiectasis/ |
| 101 | bronchiectas#s.ti,ab,kw. |
| 102 | or/89-101 [**COPD] |
| 103 | exp Heart Failure/ |
| 104 | exp Ventricular Dysfunction/ |
| 105 | ((heart or cardiac or myocardial) adj (failure or decompensation)).ti,ab,kw. |
| 106 | CHF.ti,ab,kw. |
| 107 | (ventric* adj6 (d#sfunction* or function*)).ti,ab,kw. |
| 108 | Pulmonary Edema/ |
| 109 | (pulmonary adj (edema* or oedema*)).ti,ab,kw. |
| 110 | wet lung*.ti,ab,kw. |
| 111 | or/103-110 [**Congestive Heart Failure] |
| 112 | exp Epilepsy/ |
| 113 | exp Seizures/ |
| 114 | (epilep* or seizure* or convuls*).ti,ab,kw. |
| 115 | (absence status or status epilepticus or petit mal or grand mal).ti,ab,kw. |
| 116 | Eclampsia/ |
| 117 | eclamp*.ti,ab,kw. |
| 118 | or/112-117 [**Epilepsy and seizures] |
| 119 | exp Diabetes Mellitus, Type 1/ |
| 120 | exp Diabetes Mellitus, Type 2/ |
| 121 | exp Insulin Resistance/ |
| 122 | exp Hyperglycemia/ |
| 123 | exp Diabetes Complications/ |
| 124 | (IDDM or T1DM or T1D).ti,ab,kw. |
| 125 | (insulin* depend* or insulin?depend*).ti,ab,kw. |
| 126 | ((typ? 1 or typ? I or typ?1 or typ?I) adj2 diabet*).ti,ab,kw. |
| 127 | ((acidos* or juvenil* or child* or keto* or labil* or britt*) adj2 diabet*).ti,ab,kw. |
| 128 | ((autoimmun* or sudden onset) adj2 diabet*).ti,ab,kw. |
| 129 | (insulin* defic* adj2 absolut*).ti,ab,kw. |
| 130 | (AODM or T2DM or T2D or MODY or NIDDM).ti,ab,kw. |
| 131 | (non insulin* depend* or noninsulin depend*).ti,ab,kw. |
| 132 | ((typ? 2 or typ? II or typ?2 or typ?II) adj2 diabet*).ti,ab,kw. |
| 133 | ((adult onset or maturity onset or ketosis resistant or slow onset or stable) adj2 diabet*).ti,ab,kw. |
| 134 | (diabetic adj (angiopath* or microangiopath* or vascular complication* or foot or feet or retinopath* or cardiomyopath* or coma* or acidos#s or ketoacidos#s or ketos#s or glomerulosclerosis or kidney disease* or nephropath*)).ti,ab,kw. |
| 135 | ((intracapillary or nodular) adj glomerulosclerosis).ti,ab,kw. |
| 136 | (kimmelstiel wilson adj (syndrome or disease)).ti,ab,kw. |
| 137 | (diabetic adj2 (amyotroph* or neuropath* or mononeuropath* or polyneuropath* or neuralgia*)).ti,ab,kw. |
| 138 | or/119-137 [**Diabetes and Diabetic Complications] |
| 139 | Hypertension/ |
| 140 | Essential Hypertension/ |
| 141 | hypertens*.ti,ab,kw. |
| 142 | exp Blood Pressure/ |
| 143 | ((blood pressure* or bloodpressure*) adj5 high).ti,ab,kw. |
| 144 | Hypertrophy, Left Ventricular/ |
| 145 | left ventricular hypertroph*.ti,ab,kw. |
| 146 | Heart Hypertrophy/ |
| 147 | or/139-146 [**Hypertension] |
| 148 | or/37,66,72,88,102,111,118,138,147 [**Chronic ACS Conditions] |
| 149 | Ambulatory Care/ |
| 150 | Primary Health Care/ |
| 151 | (ambulatory adj2 (care or sensitive)).ti,ab,kw. |
| 152 | ((primary adj2 (care or healthcare or health-care)) and sensitive).ti,ab,kw. |
| 153 | (ACS condition* or ACS hospitali* or ACSC or ACSCs or ACSH or ACSHs or ASSC or ASSCs or H-ACSC or H-ACSCs or AH-ACSC or AH-ACSCs or PHCSC or PHCSCs).ti,ab,kw. |
| 154 | (((avoidable or preventable or emergency or ED or unplanned or inappropriate or unnecessary or non elective or nonelective or urgent or unscheduled or unanticipated or unexpected) adj2 (hospitali* or rehospitali* or re-hospitali* or admission? or readmission? or re-admission? or admit* or readmit* or re-admit* or visit? or revisit? or re-visit? or referral?)) and (hospital* or patient? or inpatient or outpatient or emergency or ED or ambulatory)).ti,ab,kw. |
| 155 | or/149-154 [**ACS search terms] |
| 156 | 148 or 155 |
| 157 | 10 and 29 and 156 |
| 158 | randomized controlled trial.mp. |
| 159 | controlled clinical trial.mp. |
| 160 | randomized.ti,ab. |
| 161 | placebo.ti,ab. |
| 162 | or/158-161 [**RCT filter] |
| 163 | 157 not 162 |
| 164 | (Afghanistan or Angola or Albania or Armenia or Samoa or Azerbaijan or Burundi or Benin or Burkina Faso or Bangladesh or Bulgaria or Bosnia or Belarus or Belize or Bolivia or Brasil or Brazil or Bhutan or Botswana or Central African Republic or China or Cameroon or Congo or Colombia or Comoros or Cabo Verde or Costa Rica or Cuba or Djibouti or Dominica or Dominican Republic or Algeria or Ecuador or Egypt or Eritrea or Ethiopia or Fiji or Micronesia or Gabon or Ghana or Guinea or Gambia or Guinea or Grenada or Guatemala or Guyana or Honduras or Haiti or Indonesia or India or Iran or Iraq or Jamaica or Jordan or Kazakhstan or Kenya or Kyrgyz Republic or Cambodia or Kiribati or Laos or Lebanon or Liberia or Libya or Lucia or Sri Lanka or Lesotho or Morocco or Moldova or Madagascar or Maldives or Mexico or Marshall Islands or Macedonia or Mali or Myanmar or Montenegro or Mongolia or Mozambique or Mauritania or Mauritius or Malawi or Malaysia or Namibia or Niger or Nigeria or Nicaragua or Nepal or Nauru or Pakistan or Peru or Philippines or Papua New Guinea or Korea or Paraguay or Gaza or Romania or Russia or Rwanda or Sudan or Senegal or Solomon Islands or Sierra Leone or El Salvador or Somalia or Serbia or Sudan or Sao Tome or Suriname or Swaziland or Syria or Chad or Togo or Thailand or Tajikistan or Turkmenistan or Timor-Leste or Tonga or Tunisia or Turkey or Tuvalu or Tanzania or Uganda or Ukraine or Uzbekistan or Grenadines or Venezuela or Vietnam or Vanuatu or Samoa or Kosovo or Yemen or South Africa or Zambia or Zimbabwe).ti,ab,kw. [**Low and middle income countries filter] |
| 165 | 163 not 164 |
| 166 | limit 165 to (english language and yr="1990 -Current") |
| 167 | limit 166 to (article or article in press or books or "book review" or chapter) |
