# Supplementary material for: Socioeconomic inequalities in hospitalizations for chronic ambulatory care sensitive conditions: a systematic review of peer-reviewed literature, 1990–2018
Source: Int J Equity Health. 2020 May 4;19:60. doi: 10.1186/s12939-020-01160-0 (PMC7197160; doi:10.1186/s12939-020-01160-0)
Supplement: Supplementary file 3 — Additional file 3. Critical Appraisal of Included Articles (n = 31) using The Joanna Briggs Institute Critical Appraisal Tools. Study quality assessment results for each article included in the systematic review from eligible full-text articles. [file 12939_2020_1160_MOESM3_ESM.docx]

Additional File 3. Critical Appraisal of Included Articles (n=31) using The Joanna Briggs Institute Critical Appraisal Tools

| **Cohort Studies** | | | | | | | | | | | |
| --- | --- | --- | --- | --- | --- | --- | --- | --- | --- | --- | --- |
|  | The Joanna Briggs Institute Critical Appraisal Tool Questions | | | | | | | | | | |
| Citation | Were the two groups similar and recruited from the same population? | Were the exposures measured similarly to assign people to both exposed and unexposed groups? | Was the exposure measured in a valid and reliable way? | Were confounding factors identified? | Were strategies to deal with confounding factors stated? | Were the groups / participants free of the outcome at the start of the study (or at the moment of exposure)? | Were the outcomes measured in a valid and reliable way? | Was the follow up time reported and sufficient to be long enough for outcomes to occur? | Was follow up complete, and if not, were the reasons to loss to follow up described and explored? | Were strategies to address incomplete follow up utilized? | Was appropriate statistical analysis used? |
| Agabiti, N. *et al*., 2009 | Unclear | Yes | Yes | Yes | Yes | Yes | Yes | Yes | Yes | N/A | Yes |
| Aube-Maurice, J. *et al*., 2012 | Yes | Yes | Yes | Yes | Yes | Yes | Yes | Yes | Yes | N/A | Yes |
| Chen, P-C. *et al*., 2015 | Yes | Yes | Unclear | Yes | Yes | Yes | Yes | Yes | Unclear | Unclear | Yes |
| Christensen, S. *et al*., 2011 | Yes | Yes | Yes | Yes | Yes | Yes | Yes | Yes | Yes | Unclear | Yes |
| Eisner, M. *et al*., 2011 | Yes | Yes | Yes | Yes | Yes | Yes | Yes | Yes | Unclear | Unclear | Yes |
| Govan, L. *et al*., 2012 | Yes | Yes | Yes | Yes | Yes | Yes | Unclear | No | Yes | N/A | Yes |
| Li, X. *et al*., 2008 | Yes | Yes | Yes | Yes | Yes | Yes | Yes | Yes | Yes | N/A | Yes |
| Lofqvist, T. *et al*., 2014 | Yes | Yes | Yes | Yes | Yes | Yes | Yes | Yes | Yes | N/A | Yes |
| Payne, R. *et al*., 2013 | Yes | Yes | Yes | Yes | Yes | Yes | Yes | Yes | Unclear | No | Yes |
| Prescott, E. *et al*., 1999 | Unclear | Yes | Yes | Yes | Yes | Yes | Yes | Yes | Yes | N/A | Yes |
| Quan, H. *et al*., 2013 | Yes | Yes | Yes | Yes | Yes | Yes | Unclear | Yes | Yes | Unclear | Yes |
| Roos, L. *et al*., 2005 | Yes | Yes | Yes | Yes | Yes | Yes | Yes | Yes | Unclear | Unclear | Yes |
| Shah, R. *et al*., 2011 | Yes | Yes | Yes | Yes | Yes | Yes | Yes | Yes | Unclear | Unclear | Yes |
| Shulman, R. *et al*., 2018 | Yes | Yes | Yes | Yes | Yes | Yes | Yes | Yes | Yes | N/A | Yes |
| Walker, R. *et al*., 2013 | Yes | Yes | Yes | Yes | Yes | Yes | Yes | Yes | Yes | N/A | Yes |

| **Cross-sectional Studies** | | | | | | | | |
| --- | --- | --- | --- | --- | --- | --- | --- | --- |
|  | The Joanna Briggs Institute Critical Appraisal Tool Questions | | | | | | | |
| Citation | Were the criteria for inclusion in the sample clearly defined? | Were the study subjects and the setting described in detail? | Was the exposure measured in a valid and reliable way? | Were objective, standard criteria used for measurement of the condition? | Were confounding factors identified? | Were strategies to deal with confounding factors stated? | Were the outcomes measured in a valid and reliable way? | Was appropriate statistical analysis used? |
| Asaria, M. *et al*., 2016 | N/A | No | Yes | N/A | Yes | Yes | Yes | Yes |
| Bacon, S. *et al*., 2009 | Yes | Yes | Yes | Yes | Yes | Yes | Unclear | Yes |
| Banham, D. *et al*., 2010 | N/A | Yes | Yes | N/A | Yes | Yes | Yes | Yes |
| Begley, C. *et al*., 2009 | Yes | Yes | Yes | Unclear | Yes | Yes | Unclear | Yes |
| Bocour, A. *et al*., 2016 | Yes | No | Yes | N/A | Yes | Yes | Yes | Yes |
| Booth, G. *et al*., 2003 | Yes | Yes | Yes | Yes | Yes | Yes | Yes | Yes |
| Davies, S. *et al*., 2017 | Yes | No | Yes | N/A | Yes | Yes | Yes | Yes |
| Disano, J. *et al*., 2010 | Yes | No | Yes | N/A | Yes | Yes | Unclear | Yes |
| Fleetcroft, R. *et al*., 2017 | Yes | No | Yes | N/A | Yes | Yes | Yes | Yes |
| Gupta, R. *et al*., 2018 | Yes | No | Yes | N/A | Yes | Yes | Yes | Yes |
| Jackson, R. *et al*., 2001 | Yes | No | Yes | N/A | Yes | Yes | Yes | Yes |
| Lemstra, M. *et al*., 2006 | Yes | No | Yes | N/A | Yes | Yes | Yes | Yes |
| Macleod, M. *et al*., 2002 | Yes | No | Yes | N/A | Yes | Yes | Yes | Yes |
| Roberts, S. *et al*., 2012 | Yes | Yes | Yes | Yes | Yes | Yes | Yes | Yes |
| Sheringham, J. *et al*., 2017 | Yes | No | Yes | N/A | Yes | Yes | Yes | Yes |
