# Supplementary material for: Socioeconomic inequalities in hospitalizations for chronic ambulatory care sensitive conditions: a systematic review of peer-reviewed literature, 1990–2018
Source: Int J Equity Health. 2020 May 4;19:60. doi: 10.1186/s12939-020-01160-0 (PMC7197160; doi:10.1186/s12939-020-01160-0)
Supplement: Supplementary file 4 — Additional file 4. Critical Appraisal of Articles Excluded after Appraisal (n = 18) using The Joanna Briggs Institute Critical Appraisal Tools. Study quality assessment results for each article excluded upon appraisal from eligible full-text articles. [file 12939_2020_1160_MOESM4_ESM.docx]

Additional File 4. Critical Appraisal of Articles Excluded after Appraisal (n=18) using The Joanna Briggs Institute Critical Appraisal Tools

| **Cohort Studies** | | | | | | | | | | | |
| --- | --- | --- | --- | --- | --- | --- | --- | --- | --- | --- | --- |
|  | The Joanna Briggs Institute Critical Appraisal Tool Questions | | | | | | | | | | |
| Citation | Were the two groups similar and recruited from the same population? | Were the exposures measured similarly to assign people to both exposed and unexposed groups? | Was the exposure measured in a valid and reliable way? | Were confounding factors identified? | Were strategies to deal with confounding factors stated? | Were the groups / participants free of the outcome at the start of the study (or at the moment of exposure)? | Were the outcomes measured in a valid and reliable way? | Was the follow up time reported and sufficient to be long enough for outcomes to occur? | Was follow up complete, and if not, were the reasons to loss to follow up described and explored? | Were strategies to address incomplete follow up utilized? | Was appropriate statistical analysis used? |
| Eisner, M. *et al.,* 2001 (1) | Unclear | Yes | Yes | Yes | Yes | Yes | Unclear | Yes | Yes | Yes | Yes |
| Reason for exclusion of Eisner, M. *et al*., 2001: High risk of loss to follow-up bias | | | | | | | | | | | |
| Stewart, S. *et al.,* 2006 (2) | Yes | Yes | Yes | Yes | Yes | Yes | Yes | Yes | Yes | Yes | No |
| Reason for exclusion of Stewart, S. *et al*., 2006: Inappropriate use of backward step-wise selection of model variables | | | | | | | | | | | |

| **Cross-sectional Studies** | | | | | | | | |
| --- | --- | --- | --- | --- | --- | --- | --- | --- |
|  | The Joanna Briggs Institute Critical Appraisal Tool Questions | | | | | | | |
| Citation | Were the criteria for inclusion in the sample clearly defined? | Were the study subjects and the setting described in detail? | Was the exposure measured in a valid and reliable way? | Were objective, standard criteria used for measurement of the condition? | Were confounding factors identified? | Were strategies to deal with confounding factors stated? | Were the outcomes measured in a valid and reliable way? | Was appropriate statistical analysis used? |
| Ansari, Z. *et al.,* 2007 (3) | Yes | No | Yes | N/A | Yes | Yes | Yes | No |
| Reason for exclusion of Ansari, Z. *et al*., 2007: Inappropriate use of weighted least squares regression | | | | | | | | |
| Barnett, R. *et al.,* 2003 (4) | N/A | No | Yes | N/A | No | No | Unclear | No |
| Reason for exclusion of Barnett, R. *et al*., 2003: Inappropriate use of linear regression | | | | | | | | |
| Barnett, R. *et al.,* 2010 (5) | N/A | No | Yes | N/A | Yes | Yes | Unclear | No |
| Reason for exclusion of Barnett, R. *et al*., 2010: Inappropriate use of stepwise linear regression | | | | | | | | |
| Billings, J. *et al.,* 1993 (6) | Yes | No | Yes | N/A | Yes | Yes | Unclear | Unclear |
| Reason for exclusion of Billings, J. *et al*., 1993: Lacked sufficient methodological detail for critical appraisal | | | | | | | | |
| Billings, J. *et al.,* 1996 (7) | Yes | No | Yes | N/A | Yes | Yes | Unclear | Unclear |
| Reason for exclusion of Billings, J. *et al*., 1996: Lacked sufficient methodological detail for critical appraisal | | | | | | | | |
| Cable, G. *et al.,* 2002 (8) | Yes | No | Yes | N/A | Yes | Yes | Unclear | No |
| Reason for exclusion of Cable, G. *et al*., 2002: Inappropriate use of linear regression | | | | | | | | |
| Carr, W. *et al.,* 1992 (9) | Yes | No | Yes | N/A | Yes | Yes | Yes | No |
| Reason for exclusion of Carr, W. *et al*., 1992: Inappropriate use of linear regression | | | | | | | | |
| Castro, M. *et al.,* 2001 (10) | Yes | No | Yes | N/A | No | No | Yes | Unclear |
| Reason for exclusion of Castro, M. *et al*., 2001: Inappropriate methodology - Use of outdated 1940 US Standard population | | | | | | | | |
| DeLia, D. *et al*., 2003 (11) | Yes | Yes | Yes | N/A | Yes | Yes | Unclear | No |
| Reason for exclusion of DeLia, D. *et al*., 2003: Inappropriate use of linear regression | | | | | | | | |
| Dimitrovova, K. *et al*., 2017 (12) | Yes | Yes | Yes | N/A | Yes | Yes | Yes | No |
| Reason for exclusion of Dimitrovova, K. *et al*., 2017: Inappropriate use of linear regression | | | | | | | | |
| Feldman, L. *et al.,* 2004 (13) | Yes | No | Yes | N/A | Yes | Yes | Yes | No |
| Reason for exclusion of Feldman, L. *et al*., 2004: Inappropriate use of linear regression | | | | | | | | |
| Lin, S. *et al.,* 1999 (14) | Yes | No | Yes | N/A | Yes | Yes | Yes | No |
| Reason for exclusion of Lin, S. *et al*., 1999: Inappropriate use of stepwise linear regression | | | | | | | | |
| McGrath, R. *et al.,* 2011 (15) | Yes | No | Yes | N/A | Yes | Yes | Yes | No |
| Reason for exclusion of McGrath, R. *et al*., 2011: Inappropriate use of forward stepwise linear regression and modeling of income | | | | | | | | |
| Milne, B. *et al.,* 2015 (16) | Yes | No | Yes | N/A | Yes | Yes | Yes | No |
| Reason for exclusion of Milne, B. *et al*., 2015: Inappropriate methodology - Did not account for longitudinal nature of the data | | | | | | | | |
| Neudorf, C. *et al.,* 2015 (17) | Yes | No | Yes | N/A | No | No | Yes | No |
| Reason for exclusion of Neudorf, C. *et al*., 2015: Lacked sufficient methodological detail for critical appraisal | | | | | | | | |
| Ray, N. *et al.,* 1998 (18) | Yes | No | Yes | N/A | Yes | Yes | Yes | No |
| Reason for exclusion of Ray, N. *et al*., 1998: Inappropriate use of linear regression | | | | | | | | |

**References**

1. Eisner MD, Katz PP, Yelin EH, Shiboski SC, Blanc PD. Risk factors for hospitalization among adults with asthma: the influence of sociodemographic factors and asthma severity. Respiratory research. 2001;2(1):53-60.

2. Stewart S, Murphy NF, McMurray JJV, Jhund P, Hart CL, Hole D. Effect of socioeconomic deprivation on the population risk of incident heart failure hospitalisation: an analysis of the Renfrew/Paisley Study. European journal of heart failure. 2006;8(8):856-63.

3. Ansari Z, Dunt D, Dharmage SC. Variations in hospitalizations for chronic obstructive pulmonary disease in rural and urban Victoria, Australia. Respirology (Carlton, Vic). 2007;12(6):874-80.

4. Barnett R, Lauer G. Urban deprivation and public hospital admissions in Christchurch, New Zealand, 1990-1997. Health & social care in the community. 2003;11(4):299-313.

5. Barnett R, Malcolm L. Practice and ethnic variations in avoidable hospital admission rates in Christchurch, New Zealand. Health & place. 2010;16(2):199-208.

6. Billings J, Zeitel L, Lukomnik J, Carey TS, Blank AE, Newman L. Impact of socioeconomic status on hospital use in New York City. Health affairs (Project Hope). 1993;12(1):162-73.

7. Billings J, Anderson GM, Newman LS. Recent findings on preventable hospitalizations. Health affairs (Project Hope). 1996;15(3):239-49.

8. Cable G. Income, race, and preventable hospitalizations: a small area analysis in New Jersey. Journal of health care for the poor and underserved. 2002;13(1):66-80.

9. Carr W, Zeitel L, Weiss K. Variations in asthma hospitalizations and deaths in New York City. American journal of public health. 1992;82(1):59-65.

10. Castro M, Schechtman KB, Halstead J, Bloomberg G. Risk factors for asthma morbidity and mortality in a large metropolitan city. The Journal of asthma : official journal of the Association for the Care of Asthma. 2001;38(8):625-35.

11. DeLia D. Distributional issues in the analysis of preventable hospitalizations. Health services research. 2003;38(6 Pt 2):1761-79.

12. Dimitrovova K, Costa C, Santana P, Perelman J. "Evolution and financial cost of socioeconomic inequalities in ambulatory care sensitive conditions: an ecological study for Portugal, 2000-2014". International journal for equity in health. 2017;16(1):145.

13. Feldman L, McMullan C, Abernathy T. Angina and socio-economic status in Ontario: how do characteristics of the county you live in influence your chance of developing heart disease? Canadian journal of public health = Revue canadienne de sante publique. 2004;95(3):228-32.

14. Lin S, Fitzgerald E, Hwang SA, Munsie JP, Stark A. Asthma hospitalization rates and socioeconomic status in New York State (1987-1993). The Journal of asthma : official journal of the Association for the Care of Asthma. 1999;36(3):239-51.

15. McGrath R, Stransky M, Seavey J. The Impact of Socioeconomic Factors on Asthma Hospitalization Rates by Rural Classification. Journal of Community Health. 2011;36(3):495-503.

16. Milne BJ, Parker K, McLay J, von Randow M, Lay-Yee R, Hider P, et al. Primary health care access and ambulatory sensitive hospitalizations in New Zealand. The Journal of ambulatory care management. 2015;38(2):178-87.

17. Neudorf C, Fuller D, Cushon J, Glew R, Turner H, Ugolini C. An analytic approach for describing and prioritizing health inequalities at the local level in Canada: a descriptive study. CMAJ open. 2015;3(4):E366-72.

18. Ray NF, Thamer M, Fadillioglu B, Gergen PJ. Race, income, urbanicity, and asthma hospitalization in California: a small area analysis. Chest. 1998;113(5):1277-84.
